# Heart Failure Treatments Such As Angiotensin Receptor/Neprilysin Inhibitor Improve Heart Failure Status and Glucose Metabolism

**DOI:** 10.7759/cureus.22762

**Published:** 2022-03-02

**Authors:** Yusuke Kashiwagi, Tomohisa Nagoshi, Kazuo Ogawa, Makoto Kawai, Michihiro Yoshimura

**Affiliations:** 1 Division of Cardiology, Department of Internal Medicine, The Jikei University School of Medicine, Tokyo, JPN

**Keywords:** glucose metabolism, islet beta-cell function, insulin resistance, heart failure with reduced ejection fraction, angiotensin receptor/neprilysin inhibitor

## Abstract

A recent study suggested that angiotensin receptor/neprilysin inhibitor (ARNI; sacubitril/valsartan) can improve functional capacity and cardiac reverse remodeling in patients with heart failure with reduced ejection fraction (HFrEF). Another study suggested that ARNI reduced glycated hemoglobin (HbA1c) in patients with diabetes and HFrEF; however, the details of its efficacy are unknown. We herein report a case of HFrEF with abnormal glucose metabolism in which ARNI was initiated. On the 7^th^ day of admission (before the initiation of ARNI), blood tests showed an abnormal glucose metabolism as follows: fasting blood glucose 134 mg/dL; and fasting blood insulin 11.4 µU/mL (homeostasis model assessment of insulin resistance (HOMA-IR) index 3.77; homeostasis model assessment of β-cell function (HOMA-β), 57.8%). On the 23^rd^ day after the initiation of ARNI, even though the patient was not using hypoglycemic drugs, his fasting blood glucose markedly decreased to 70 mg/dL without hypoglycemic symptoms, and his fasting blood insulin decreased to 5.4 µU/mL (HOMA-IR decreased to 0.93, HOMA-β increased to 277.7%). These results imply that ARNI has the potential to improve insulin resistance and the islet beta-cell function in patients with heart failure, in addition to the original effect of improving the hemodynamics, although the effect of improving the glucose metabolism is also considered to have been influenced by the improvement of the heart failure status and other drugs that the patient was taking.

## Introduction

Patients with heart failure with reduced ejection fraction (HFrEF) are at high risk for disease progression, hospitalization, and mortality. Angiotensin-converting enzyme (ACE) inhibitors have long been the cornerstone of treatment for heart failure, including HFrEF [1]. A recent study showed that angiotensin receptor/neprilysin inhibitor (ARNI; sacubitril/valsartan) treatment resulted in a lower rate of hospitalization for heart failure or death from cardiovascular causes in comparison to ACE inhibitor treatment among patients with HFrEF [2].

A previous study suggested that ARNIs can improve the functional capacity and cardiac reverse remodeling in patients with HFrEF, and that this anti-remodeling effect may lead to the prevention of ventricular arrhythmia and even sudden death [3]. In addition, a previous study showed that patients with diabetes and HFrEF who received ARNI had a greater long-term reduction in HbA1c than those who received ACE inhibitors [4]. Another study reported in patients with heart failure, ARNI decreases fructosamines that reflect average glucose levels over the preceding two to three weeks [5]. Although it has been suggested that ARNI may have an effect on the improvement of derangement of glucose metabolism, its detailed efficacy has not been fully clarified. We herein report a case of HFrEF in which insulin resistance and the islet beta-cell function were significantly improved early after the initiation of ARNI.

## Case presentation

**Figure 1:**
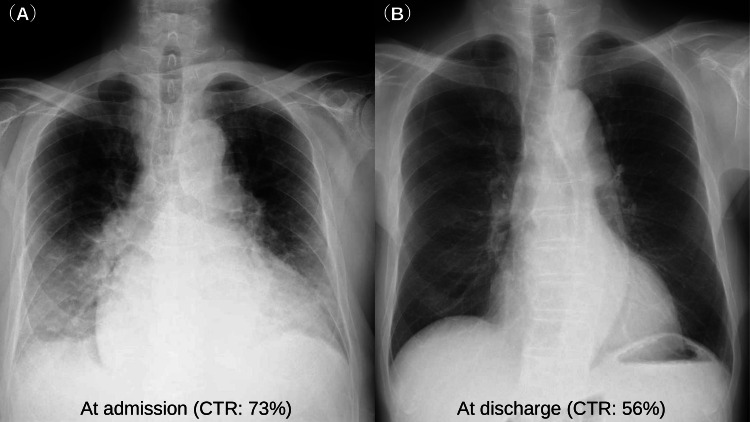
Chest X-ray at the time of admission and discharge. Chest X-ray showed cardiomegaly (CTR: 73%) and pulmonary congestion at the time of admission (A). The CTR decreased to 56% and pulmonary congestion disappeared at the time of discharge (B). CTR: cardiothoracic ratio

**Figure 2:**
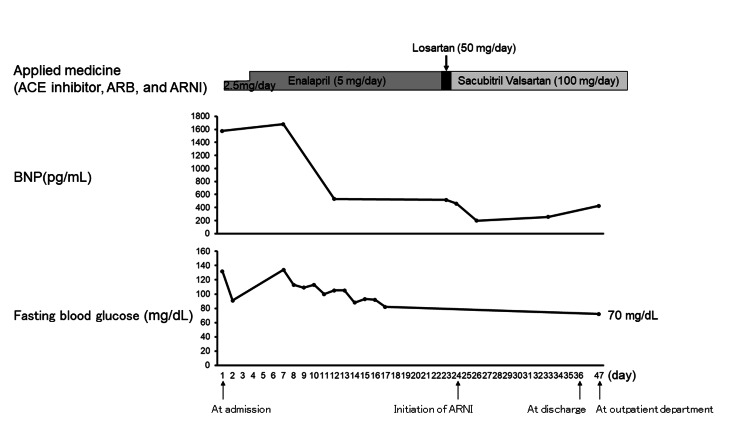
The course of medical treatment (ACE inhibitor, ARB, and ARNI), BNP level, and the fasting blood glucose level. On the 23rd day of admission, enalapril (5 mg, daily) was switched to losartan (50 mg, daily). The next day, losartan was switched to sacubitril/valsartan (50 mg, twice daily). In the outpatient department on the 11th day after discharge (23 days after the initiation of ARNI treatment), blood tests showed that the BNP level decreased to 425 pg/mL, and the fasting blood glucose level decreased to 70 mg/dL. ACE: angiotensin-converting enzyme; ARB: angiotensin II receptor blocker; ARNI: angiotensin receptor/neprilysin inhibitor; BNP: B-type natriuretic peptide

A 76-year-old man without a history of hospital visits for medical illness, presented with bilateral leg edema that persisted for 2 months, and exertional breathlessness and orthopnea that had persisted for 2 weeks. On admission, a physical examination revealed the following: blood pressure 134/106 mmHg; heart rate 101 bpm; body temperature 36.4ºC; and saturation of percutaneous oxygen 97% on room air. A 12-lead electrocardiogram revealed sinus rhythm at 100 bpm with normal intervals, left axis deviation, and flattening of the T wave in I, aVL, V5, and V6. Chest X-ray showed cardiomegaly (cardiothoracic ratio (CTR): 73%) and pulmonary congestion (Figure 1A). Transthoracic echocardiography (TTE) showed diffuse left ventricular hypokinesis with an ejection fraction (EF) of 25-30% and mild valvular disease. Blood tests showed that the level of B-type natriuretic peptide (BNP) increased to 1576 pg/mL, and there was no obvious anemia or iron deficiency (hemoglobin 14.5 g/dL; serum iron 112 μg/dL; unsaturated iron-binding capacity (UIBC), 169 μg/dL; total iron-binding capacity (TIBC) 281 μg/dL; and ferritin 139 ng/ml). Based on these results, the patient was diagnosed with HFrEF, and treatment with diuretics was started. Coronary angiography showed no significant coronary stenosis, and cardiac magnetic resonance imaging (MRI) showed no specific findings suggestive of any myocardiopathy. Although the etiology of heart failure was unknown, the possibility that the diagnosis of the present case was dilated cardiomyopathy could not be ruled out. On the 7th day of admission, blood tests showed the following: hemoglobin A1c (HbA1c) 6.3%; fasting blood glucose 134 mg/dL; and fasting blood insulin 11.4 µU/mL (homeostasis model assessment of insulin resistance (HOMA-IR) index 3.77; homeostasis model assessment of β-cell function (HOMA-β), 57.8%) (Table 1). On the 23rd day of admission, enalapril (5 mg, daily) was switched to losartan (50 mg, daily), and the next day, losartan was switched to sacubitril/valsartan (50 mg, twice daily) (Figure 2). Eventually, TTE showed no significant change in EF, the CTR on chest X-ray decreased to 56% (Figure 1B) and the patient was discharged on the 36th day of admission. The prescriptions at discharge were as follows: eplerenone (50 mg, daily), amiodarone (100 mg, twice daily), bisoprolol fumarate (0.625 mg, daily), pimobendan (2.5 mg twice, daily), apixaban (5 mg, twice daily), tolvaptan (7.5 mg, daily), sacubitril valsartan sodium hydrate (50 mg, twice daily), and furosemide (80 mg, daily). When examined in the outpatient department on the 11th day after discharge (23 days after the initiation of ARNI), the patient’s condition was stable without a worsening of heart failure, and a blood test showed that the level of BNP had decreased to 425 pg/mL. Even though the patient was not using hypoglycemic drugs, his fasting blood glucose decreased to 70 mg/dL without hypoglycemic symptoms, and his fasting blood insulin decreased to 5.4 µU/mL (HOMA-IR decreased to 0.93, HOMA-β increased to 277.7%) (Table 1). His insulin resistance and islet beta-cell function were markedly improved early after the initiation of ARNI treatment.

 

**Table 1:** Comparison of glucose metabolism-related parameters before and after ARNI treatment. ARNI: angiotensin receptor/neprilysin inhibitor; SBP: systolic blood pressure; DBP: diastolic blood pressure; HOMA-IR: homeostasis model assessment of insulin resistance; HOMA-β: homeostasis model assessment of β-cell function; HDL: high-density lipoprotein cholesterol; LDL: low-density lipoprotein cholesterol

	Before the beginning of ARNI (On 7-day admission)	After the ARNI (23 days after the initiation of ARNI)
Body weight （kg）	79.5	64.5
SBP/DBP (mmHg)	92/72	110/60
Heart rate (beats per minute)	80	58
B-type natriuretic peptide (pg/mL)	1681	425
Fasting blood glucose(mg/dL)	134	70
Fasting blood insulin (µU/mL)	11.4	5.4
HOMA-IR	3.77	0.93
HOMA-β (%)	57.8	277.7
Hemoglobin A1c (%)	6.3	6.4
HDL (mg/dL)	69	80
LDL (mg/dL)	114	128
Triglyceride (mg/dL)	117	93

## Discussion

We described a case of HFrEF in which insulin resistance and the islet beta-cell function were significantly improved early after the initiation of ARNI treatment, although the effect of improving the glucose metabolism is also considered to have been influenced by the improvement of the heart failure status and other drugs that the patient was taking. This result implied that the administration of ARNI may improve the abnormal glucose metabolism in patients with HFrEF.

Diabetes is an important risk factor for the development of heart failure. Conversely, the severity of heart failure is also strongly related to the development of diabetes [6]. Previous reports suggested that inhibitors of the renin-angiotensin system (RAS), such as ACE inhibitors and angiotensin II receptor blockers (ARBs), may reduce the incidence of the development of diabetes [7]. Glucose-induced RAS activation increases oxidative stress, tissue inflammation, cell proliferation, and apoptosis [8]. Furthermore, the inhibition of RAS prevents these deleterious effects of hyperglycemia and improves the beta-cell function [9]. In addition, RAS blockade promotes glucose utilization in skeletal muscle, reduces adipocyte size, and improves adipose tissue function, leading to the improvement of insulin resistance [10]. Moreover, glucagon-like peptide-1 (GLP-1), a neuropeptide of the incretin family and potent antihyperglycemic hormone, is partially degraded by neprilysin [11]. Consequently, the inhibition of neprilysin increases GLP-1 levels, reduces plasma dipeptidyl peptidase-4 (DPP-4) activity, and improves the beta-cell function [12]. Natriuretic peptides, which are increased by neprilysin inhibition, accelerate lipolysis in adipose tissue and have the potential to suppress the progression of diabetes by improving insulin resistance [13]. Indeed, it was previously shown that exogenous A-type natriuretic peptide (ANP) administration in mice fed with high-fat diet showed significantly improvement in insulin resistance by attenuating hepatic steatosis and inducing adipose tissue browning [14].

A previous study reported that, in congestive heart failure patients, the addition of ARB to ACE inhibitor significantly decreased fasting insulin levels and HOMA-IR values at 16 weeks, but fasting blood glucose levels did not change significantly [15]. Another report showed that, in non-diabetic patients, HOMA-IR values decreased by 1.2-1.6 within 16 weeks of starting treatment with ARB [16]. In the present case, the fasting blood insulin decreased to 5.4 µU/mL from 11.4 µU/mL, and HOMA-IR prominently decreased by 2.84 at the timing of 23 days after the initiation of ARNI. Furthermore, fasting blood glucose also markedly decreased to 70 mg/dL from 134 mg/dL. Therefore, ARNI, which is a combination drug consisting of an ARB and a neprilysin inhibitor, is considered to have a substantial effect on improving the derangement of glucose metabolism. In general, abnormal glucose metabolism (e.g., insulin resistance) is prevalent among patients with heart failure [17]. In addition, a state of stress due to hospitalization for heart failure could cause the deterioration of the glucose metabolism due to activation of the sympathetic nervous system and the increased secretion of stress hormones such as epinephrine and cortisol [18]. Therefore, it is considered that the improvement of the abnormal glucose metabolism must also have been influenced by the improvement of heart failure status. Furthermore, eplerenone, which was taken at the time of discharge, is a RAS inhibitor. In addition to the effect of ARNI, the improvement of the abnormal glucose metabolism may have been strengthened by eplerenone.

In patients with heart failure, anemia adversely affects the hemodynamics, and parenteral iron replacement therapy was shown to improve subjective symptoms and exercise capacity [19]. A previous report showed that intravenous iron infusion improved abnormal glucose metabolism in patients with type 2 diabetes and iron deficiency [20]. In this case, there was no obvious anemia or iron deficiency during the course of hospitalization, so iron transfusion was not performed.

## Conclusions

In conclusion, we encountered a case of HFrEF in which insulin resistance and the islet beta-cell function significantly improved after the initiation of ARNI treatment, although the effect of improving the glucose metabolism is considered to have also been influenced by the improvement of the heart failure status and other drugs (e.g., eplerenone) that the patient was taking. Therefore, in heart failure patients with diabetes or impaired glucose tolerance, ARNI may improve derangement of the glucose metabolism, in addition to the original effect of improving the hemodynamics.
